# Nutritional status and out-of-hospital mortality in vascular surgery patients

**DOI:** 10.1371/journal.pone.0270396

**Published:** 2022-07-21

**Authors:** G. C. I. von Meijenfeldt, K. M. Mogensen, M. J. van der Laan, C. J. Zeebregts, K. B. Christopher

**Affiliations:** 1 Division of Vascular Surgery, Department of Surgery, University Medical Center Groningen, University of Groningen, Groningen, The Netherlands; 2 Department of Surgery, Deventer Ziekenhuis, Deventer, The Netherlands; 3 Department of Nutrition, Brigham and Women’s Hospital, Boston, Massachusetts, United States of America; 4 Renal Division, Brigham and Women’s Hospital, Channing Division of Network Medicine, Boston, Massachusetts, United States of America; Technion - Israel Institute of Technology, ISRAEL

## Abstract

**Background:**

Malnutrition is often present in vascular surgery patient during hospital admission. The present evidence of the consequence malnutrition has on morbidity and mortality is limited.

**Aim:**

The purpose of this study was to determine the effect of nutritional status on out-of-hospital mortality in vascular surgery patients.

**Methods:**

An observational cohort study was performed, studying non-cardiac vascular surgery patients surviving hospital admission 18 years or older treated in Boston, Massachusetts, USA. The exposure of interest was nutritional status categorized as well nourished, at-risk for malnutrition, nonspecific malnutrition or protein-energy malnutrition. The all cause 90-day mortality following hospital discharge was the primary outcome. Adjusted odds ratios were estimated by multivariable logistic regression models.

**Results:**

This cohort included 4432 patients comprised of 48% women and a mean age 61.7 years. After evaluation by a registered dietitian, 3819 patients were determined to be well nourished, 215 patients were at-risk for malnutrition, 351 had non-specific malnutrition and 47 patients had protein-energy malnutrition. After adjustment for age, sex, ethnicity, medical versus surgical Diagnosis Related Group type, Deyo-Charlson index, length of stay, and vascular Current Procedural Terminology code category, the 90-day post-discharge mortality odds ratio for patients with non-specific malnutrition OR 1.96 (95%CI 1.21, 3.17) and for protein-energy malnutrition OR 3.58 (95%CI 1.59, 8.06), all relative to patients without malnutrition.

**Discussion:**

Nutritional status is a strong predictor of out-of-hospital mortality. This suggests that patient with vascular disease suffering from malnutrition could benefit from more intensified In-hospital and out-of-hospital dietary guidance and interventions.

## Introduction

Malnutrition in hospitalized patients is common and associated with adverse outcomes yet is undervalued and often underreported [1–7]. Malnutrition in general and subspecialty surgical patients is an important predictor of increased hospital stay, major complications, hospital readmissions and mortality [8–11]. Adverse outcomes associated with malnutrition are heightened the critically ill and elderly patients [12–14].

Data describing the effects of malnutrition on mortality and other adverse events in vascular surgery patients are limited to small studies [6, 15, 16]. Patients with pre-operative protein energy depletion more frequently develop systemic inflammatory response syndrome (SIRS) following major vascular surgery especially after open vascular surgery [17]. In amputation patients, known for their high pre-existing co-morbidities, pre-operative hypoalbuminemia is associated with increased perioperative mortality [16]. This association is also known for aortic aneurysm patients [18]. As albumin is also a negative acute phase reactant it may be more an indicator of inflammation rather than malnutrition directly as the inflammation is a contributor to the development of malnutrition. Existing malnutrition studies in vascular surgery focus on in-hospital outcomes. Outcomes in survivors of hospitalization are unexplored in vascular surgery patients.

Therefore we performed an observational study on inpatients who underwent vascular surgery and survived hospitalization. We utilized data determined by a registered dietitian (RD) evaluation to study the association of nutrition status and post-discharge outcomes. We hypothesized that patients who underwent vascular surgery and survived hospitalization, malnutrition would independently be associated with mortality and other adverse events following hospital discharge.

## Materials and methods

### Source population

Administrative and laboratory data were extracted from individuals admitted to Brigham and Women’s Hospital (BWH) in Boston, Massachusetts, USA. BWH is a 793 bed primary and tertiary care facility that provides full spectrum vascular surgery to an ethnically and socioeconomically diverse population within eastern Massachusetts and the surrounding area.

### Data sources

Data on eligible patients were obtained through the Research Patient Data Registry (RPDR) between 2004 and 2012. The RPDR is a computerized registry that serves as a central data warehouse for all inpatient and outpatient records at Partners HealthCare sites [19, 20] including the BWH and Massachusetts General Hospital (MGH). The RDPR has been used for other clinical research studies, and mortality and coding data from the RPDR have been validated [5, 21]. Since 2004, the Department of Nutrition at the BWH has collected inpatient nutrition information in an electronic data capture system. The nutrition status evaluations are performed by RDs who collect data related to energy and protein intake, wasting of muscle mass and subcutaneous fat as well as weight loss [22]. Approval for the study was granted by the Partners Human Research Committee (Institutional Review Board). Requirement for individual patient consent was waived as the data were analysed anonymously.

### Study population

Patients eligible for inclusion were hospitalized adults aged ≥18 years who were admitted to intensive care unit (ICU) or in-patient care ward and who underwent non-cardiac vascular surgery during their hospitalization and survived to hospital discharge. All patients were assigned at least one Current Procedural Terminology (CPT) code for vascular surgery [23] (S1 File), and were assigned a Diagnostic Related Group (DRG) code. Patients treated with open vascular surgery as well as endovascular procedures were included. In the analytic cohort we excluded patients who died in hospital, did not have vascular surgery related CPT codes assigned, a DRG code assigned or had missing data for confounding variables (age, sex, ethnicity, length of stay). Between 2004 and 2012, there were 4432 patients in the analytic cohort who met these inclusion criteria.

### Exposure of interest and comorbidities

Malnutrition is diagnosed at BWH by an RD based on patient level data related to insufficient nutrient intake of energy or protein, wasting of muscle mass, and subcutaneous fat and unintentional weight loss [5, 9, 22]. In short, RDs screen all vascular surgery patients and those who are at risk are further evaluated with a formal structured objective assessment using clinical judgement and on data related to inadequate nutrient intake of energy and/or protein, wasting of muscle mass and/or subcutaneous fat and unintentional weight loss. Nutrient intake of energy and protein are determined by calorie counts of oral intake, documented intake of tube feedings and intravenous nutrient sources (dextrose, propofol, and parenteral nutrition). Energy needs are determined by the following: for BMI < 30 kg/m^2^, basal metabolic rate is calculated based on body surface area [24] and age, then activity and stress factors are applied based on severity of illness (patients are generally fed between 30–35 kcal/kg). For BMI 30–35 kg/m^2^ adjusted weight for obesity is calculated and used to calculate the basal metabolic rate based on body surface area and age, then activity and stress factors are applied based on severity of illness, similar to the approach for patients with a BMI < 30 kg/m^2^ (these patients are generally fed between 25–35 kcal/kg). Critically ill patients with BMI 35–50 kg/m^2^ are fed at 14 kcal/kg dry weight, and those with BMI > 50 kg/m^2^ are fed at 25 kcal/kg ideal body weight [25–27].

Nutrition diagnoses were categorized a priori into malnutrition absent, at risk for malnutrition, nonspecific malnutrition, or any protein-energy malnutrition [22]. Patients were categorized as nonspecific malnutrition if the patient had known risk factors (inadequate nutrient intake of energy, protein, and micronutrients) with metabolic stress (increased calorie requirement) and/or overt signs of malnutrition (wasting of muscle mass and/or subcutaneous fat) without supporting anthropometric or biochemical data present. Metabolic stress factors are utilized from prior work which measured disease specific energy expenditure relative to the predicted energy expenditure via the Harris-Benedict equation [28]. To be categorized as protein-energy malnutrition, patients must have a combination of disease-related weight loss, underweight status based on percent ideal body weight [29], overt muscle wasting, peripheral oedema, inadequate energy, or protein intake. Serum albumin, total lymphocyte count, and transferrin are part of the malnutrition criteria but RDs are trained to consider these as invalid markers of nutrition in patients with significant inflammation, altered volume status, and other conditions where these markers would be altered as a result of illness. Malnutrition was categorized as absent if patients were diagnosed as well-nourished and not at risk for malnutrition. For this study, malnutrition was considered present if the patient was diagnosed by an RD with non-specific malnutrition, mild protein-energy malnutrition, moderate protein-energy malnutrition, severe protein-energy malnutrition, marasmus, or kwashiorkor. The criteria per malnutrition category is listed in S1 File.

DRG type was defined as medical or surgical and incorporates the Diagnostic Related Grouping (DRG) methodology, devised by the Centres for Medicare & Medicaid Services and is reflective of case mix and resource utilization [21–23, 30]. Ethnicity was either self-determined or designated by a patient representative/healthcare proxy. We utilized validated International Classification of Diseases, Ninth Revision (ICD-9) coding algorithms to derive the Deyo-Charlson index comorbidity score to assess the burden of chronic illness for each patients [31–33]. We used the Healthcare Cost and Utilization Project Clinical Classification Software (CCS) multi diagnosis categories to determine diabetes, hypertension, congestive heart failure, chronic obstructive pulmonary disease, cirrhosis, and metastatic malignancy [34]. For the Deyo-Charlson index and the CCS data, chronic diagnoses were determined by ICD-9 codes assigned as outpatients or inpatients. Patients considered Emergent were admitted to the hospital via the emergency room while non-Emergent patients were admitted to the hospital following referral from an outpatient clinic or another facility. CPT code assignment was determined from dated daily clinician billing and defined according to the CPT code set maintained and published yearly by the American Medical Association [35–38]. A total of 162 vascular surgery related CPT codes were combined into Vascular Surgery Procedure Code Categories (S2 File).

### End points

The primary end point was 90-day all-cause mortality following hospital admission. Information on mortality was obtained through the Social Security Administration Death Master File which has previously been validated in our dataset for in-hospital and out-of-hospital mortality [21]. Death Master File data indicated that one hundred percent of the parent and analytic cohorts had vital status (alive or deceased) determined at 365 days following hospital discharge. The censoring date was December 31, 2013, and 100% of the parent and analytic cohorts had at least 90-day mortality follow-up after hospital discharge.

Secondary outcomes were unplanned 30-day hospital readmission to the BWH or MGH, discharge to a care facility and 365-day mortality. Thirty-day hospital readmission was determined from RPDR hospital admission data as previously described [39] and defined as a subsequent or unscheduled admission to BWH or MGH within 30 days of discharge following the hospitalization associated with the vascular surgery or intervention [23, 40, 41]. We excluded 73 readmissions with DRG codes that are commonly associated with planned readmissions in addition to DRGs for transplantation, procedures related to pregnancy, and psychiatric issues [39, 40].

### Power calculations and statistical analysis

Based on prior studies [5, 39, 42, 43], we assumed that *90-day post-discharge hospital mortality* would increase a relative 50% in patients with malnutrition (7.5%) compared to those without malnutrition (5%). With an alpha error level of 5% and a power of 80%, the minimum sample size thus required for our primary end point is 4,096 total patients.

Categorical covariates were described by frequency distribution and compared across nutrition status groups using contingency tables and chi-square testing. Continuous covariates were examined graphically and in terms of summary statistics and compared across nutrition status groups using one-way analysis of variance (ANOVA). Unadjusted associations between nutrition status groups and outcomes were estimated by bivariable logistic regression analysis. Adjusted odds ratios [44] were estimated by multivariable logistic regression models with inclusion of covariate terms thought to plausibly interact with both nutrition status and mortality. Overall model fit was assessed using the Hosmer-Lemeshow (HL) test. The performance of the model was assessed by the area under the receiver operating curve. Analyses based on fully adjusted models were performed to evaluate the malnutrition-mortality association, and P-interaction was determined to explore for any evidence of effect modification. All P values presented are 2-tailed; values below .05 were considered nominally significant. All analyses were performed using STATA 14.2MP (StataCorp LP, College Station, TX).

## Results

In Table 1 the characteristics of the 4432 patient analytic cohort were stratified according to 90-day post-discharge mortality. Most patients were men (52%), white (82%) with a mean (SD) age of 61.7 (16.7) years. The 90, 180 and 365-day post-discharge mortality rates were 3.6%, 5.9% and 9.1%, respectively. After hospitalization, 62.8% were discharged home. The 30 day hospital readmission rate was 11.5%. The median [IQR] in-hospital length of stay was 5 [1, 11] days. Factors that were associated with increased 90-day post-discharge mortality included higher age, medical DRG type, higher Deyo-Charlson index, diabetes, congestive heart failure, chronic obstructive pulmonary disease, cirrhosis, metastatic malignancy, chronic kidney disease, nutritional status, increased length of stay in hospital, discharge to facility and 30-day hospital readmission (Table 1). Vascular Surgery Procedure Code Categories associated with increased 90-day post-discharge mortality were embolectomy or thrombectomy, graft excision, major amputation, and blood vessel repair (S1 Table).

**Table 1:** Characteristics and unadjusted association of potential prognostic determinants of 90-day post discharge mortality^a^ (n = 4,432).

Characteristic	Alive^a^	Expired	Total	P-value	OR of 90-day post-discharge mortality (95%CI)
N	**4271**	**161**	**4432**		
Age-Mean ± SD	**61.2 ± 16.6**	**72.9 ± 14.9**	**61.7 ± 16.7**	**<0.001** ^†^	**1.06 (1.04, 1.07)**
Female Sex-No.(%)	**2043 (48)**	**70 (43)**	**2113 (48)**	**0.28**	**0.84 (0.61, 1.15)**
Ethnicity				**0.59**	
White-No.(%)	**3498 (82)**	**132 (82)**	**3630 (82)**		**1.00 (Referent)**
Asian-No.(%)	**73 (2)**	**1 (1)**	**74 (2)**		**0.36 (0.05, 2.63)**
Black-No.(%)	**255 (6)**	**10 (6)**	**265 (6)**		**1.04 (0.54, 2.00)**
Hispanic-No.(%)	**122 (3)**	**2 (1)**	**124 (3)**		**0.43 (0.11, 1.78)**
Not Recorded-No.(%)	**247 (6)**	**11 (7)**	**258 (6)**		**1.18 (0.63, 2.21)**
Other-No.(%)	**71 (2)**	**5 (3)**	**76 (2)**		**1.74 (0.69, 4.38)**
Non-White Ethnicity-No.(%)	**773 (18)**	**29 (18)**	**802 (18)**	**0.98**	**0.99 (0.66, 1.50)**
Medical DRG Type-No.(%)	**702 (16)**	**48 (30)**	**750 (17)**	**<0.001**	**2.16 (1.53, 3.06)**
Deyo-Charlson Index-Mean ± SD	**2.7 ± 2.4**	**4.8 ± 2.8**	**2.8 ± 2.5**	**<0.001** ^**†**^	**1.28 (1.22, 1.34)**
Diabetes-No.(%)	**929 (22)**	**49 (30)**	**978 (22)**	**0.009**	**1.57 (1.12, 2.22)**
Hypertension-No.(%)	**1832 (43)**	**73 (45)**	**1905 (43)**	**0.54**	**1.10 (0.81, 1.52)**
Congestive Heart Failure-No.(%)	**653 (15)**	**62 (39)**	**715 (16)**	**<0.001**	**3.47 (2.50, 4.82)**
Chronic Obstructive Pulmonary Disease-No.(%)	**243 (6)**	**22 (14)**	**265 (6)**	**<0.001**	**2.62 (1.64, 4.19)**
Cirrhosis-No.(%)	**24 (1)**	**7 (4)**	**31 (1)**	**<0.001**	**8.04 (3.41, 18.95)**
Metastatic Malignancy-No.(%)	**48 (1)**	**5 (3)**	**53 (1)**	**0.023**	**0.82 (1.11, 7.18)**
Chronic Kidney Disease-No.(%)	**248 (6)**	**21 (13)**	**269 (6)**	**<0.001**	**2.43 (1.51, 3.92)**
Endovascular-No.(%)	**2013 (47)**	**85 (53)**	**2098 (47)**	**0.16**	**1.26 (0.92, 1.72)**
Emergent Hospital Admission-No.(%)	**858 (20)**	**41 (25)**	**899 (20)**	**0.096**	**1.36 (0.95, 1.95)**
Nutrition Status				**<0.001**	
No malnutrition-No.(%)	**3704 (87)**	**115 (71)**	**3819 (86)**		**1.00 (Referent)**
At risk for malnutrition-No.(%)	**206 (5)**	**9 (6)**	**215 (5)**		**1.41 (0.70, 2.81)**
Non-specific malnutrition-No.(%)	**324 (8)**	**27 (17)**	**351 (8)**		**2.68 (1.74, 4.14)**
Protein-energy malnutrition-No.(%)	**37 (1)**	**10 (6)**	**47 (1)**		**8.71 (4.23, 17.93)**
Length of Stay days-Median [IQR]	**5 [1, 11]**	**12 [6, 22]**	**5 [1, 11]**	**<0.001** ^**‡**^	**1.02 (1.01, 1.02)**
Discharge to Home-No.(%)	**2738 (64)**	**47 (29)**	**2785 (63)**	**<0.001**	**0.23 (0.16, 0.33)**
30-day Readmission-No.(%)	**463 (11)**	**47 (29)**	**510 (12)**	**<0.001**	**3.39 (2.38, 4.83)**

Data presented as No. (%) unless otherwise indicated. P determined by chi-square except for † determined by ANOVA or ‡ determined by Kruskal-Wallis test.

a. Expired within 90-days following hospital discharge.

Patient characteristics were stratified according to nutritional status categories (Table 2). Significant differences were observed in patient age, ethnicity, DRG type, Deyo-Charlson index, congestive heart failure, chronic obstructive pulmonary disease, chronic kidney disease, emergent hospitalization, hospital length of stay, and 30 day readmission with respect to nutrition status categories. Details on Vascular Surgery Procedure Code Categories relative to nutrition status categories are presented in S2 Table.

**Table 2:** Characteristics of the study cohort stratified by nutritional status (n = 4,432).

Characteristic	No malnutrition	At risk for malnutrition	Non-specific malnutrition	Protein-energy malnutrition	P-value
N	**3,819**	**215**	**351**	**47**	
Age-Mean ± SD	**62 ± 16.5**	**58.9 ± 17.2**	**59.3 ± 17.8**	**64.4 ± 17.7**	**0.001†**
Female Sex-No.(%)	**1821 (48)**	**110 (51)**	**161 (46)**	**21 (45)**	**0.64**
Ethnicity					**0.010**
White-No.(%)	**3144 (82)**	**159 (74)**	**289 (82)**	**38 (81)**	
Asian-No.(%)	**61 (2)**	**5 (2)**	**6 (2)**	**2 (4)**	
Black-No.(%)	**221 (6)**	**19 (9)**	**23 (7)**	**2 (4)**	
Hispanic-No.(%)	**93 (2)**	**15 (7)**	**15 (4)**	**1 (2)**	
Not Recorded-No.(%)	**227 (6)**	**15 (7)**	**13 (4)**	**3 (6)**	
Other-No.(%)	**73 (2)**	**2 (1)**	**5 (2)**	**1 (2)**	
Non-White Ethnicity-No.(%)	**675 (18)**	**56 (26)**	**62 (18)**	**9 (19)**	**0.021**
Medical DRG Type-No.(%)	**226 (6)**	**17 (8)**	**14 (4)**	**8 (17)**	**<0.001**
Deyo-Charlson Index-Mean ± SD	**2.6 ± 2.4**	**4.0 ± 2.7**	**3.7 ± 2.8**	**5.1 ± 3.3**	**<0.001** †
Diabetes-No.(%)	**844 (22)**	**46 (21)**	**78 (22)**	**10 (21)**	**0.99**
Hypertension-No.(%)	**1669 (44)**	**78 (36)**	**136 (39)**	**22 (47)**	**0.055**
Congestive Heart Failure-No.(%)	**551 (14)**	**52 (24)**	**99 (28)**	**13 (28)**	**<0.001**
Chronic Obstructive Pulmonary Disease-No.(%)	**226 (6)**	**17 (8)**	**14 (4)**	**8 (17)**	**0.003**
Cirrhosis-No.(%)	**21 (1)**	**4 (2)**	**6 (2)**	**0 (0)**	
Metastatic Malignancy-No.(%)	**40 (1)**	**4 (2)**	**7 (2)**	**2 (4)**	**0.068**
Chronic Kidney Disease-No.(%)	**201 (5)**	**18 (8)**	**37 (11)**	**13 (28)**	**<0.001**
Endovascular-No.(%)	**1806 (47)**	**115 (53)**	**157 (45)**	**20 (43)**	**0.20**
Emergent Hospital Admission-No.(%)	**690 (18)**	**74 (34)**	**124 (35)**	**11 (23)**	**<0.001**
Length of Stay days-Median [IQR]	**4 [1, 8]**	**17 [11, 23]**	**19 [14, 28]**	**17 [10, 27]**	**<0.001** ^**‡**^
Discharge to Home-No.(%)	**1722 (45)**	**20 (9)**	**21 (6)**	**4 (9)**	**<0.001**
30-day Readmission-No.(%)	**394 (10)**	**38 (18)**	**67 (19)**	**11 (23)**	**<0.001**

Data presented as No. (%) unless otherwise indicated. P determined by chi-square except for

† determined by ANOVA or

‡ determined by Kruskal-Wallis test.

**Fig 1:**
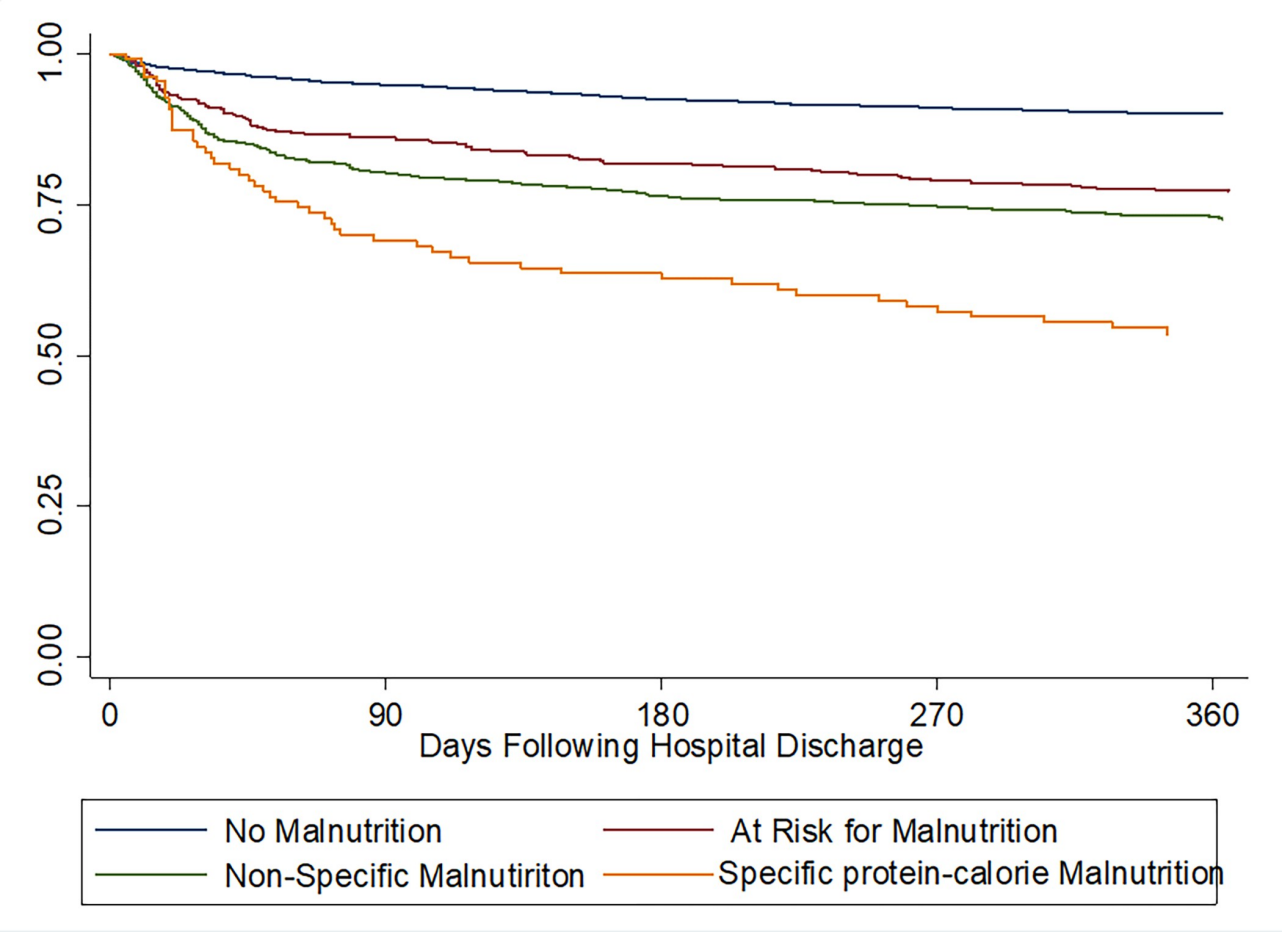
Time-to-event curves for mortality. Unadjusted mortality rates were calculated with Kaplan-Meier methods and compared with the log-rank test. Categorization of nutrition groups is per the primary analysis. The global comparison log rank P value is <0.001, indicating significantly different survival patterns.

In the analytic cohort (N = 4432) comprising survivors of hospitalization, nutrition status was a significant predictor mortality 90 days following hospital discharge (Fig 1, Table 3). Nutritional status remained a significant predictor of 90-day post-discharge mortality after adjustment for age, sex, ethnicity, Deyo-Charlson Index, DRG type, length of stay and vascular procedure code category (Table 3, Model 3). The adjusted odds of 90-day post-discharge mortality in patients with nonspecific malnutrition or protein-energy malnutrition were 2.0-fold and 3.6-fold higher, respectively, relative to patients without malnutrition. The adjusted 90-day post-discharge mortality model showed good calibration (Hosmer-Lemeshow χ^2^ 8.4, P = 0.40) and good discrimination for 90-day post-discharge mortality (c-statistic = 0.83, 95% CI, 0.80–0.87).

**Table 3:** Unadjusted and adjusted associations between nutrition status and 90-day post-discharge mortality (n = 4,432).

	Nutrition Status			
	No malnutrition^a^	At risk for malnutrition	Non-specific malnutrition	Protein-energy malnutrition
*90-day Post-Discharge Mortality*				
Crude	**1.00 (Referent)**	**1.41 (0.70, 2.81) P = 0.334**	**2.68 (1.74, 4.14) P<0.001**	**8.71 (4.23, 17.93) P<0.001**
Adjusted^b^	**1.00 (Referent)**	**1.01 (0.49, 2.08) P = 0.970**	**1.95 (1.22, 3.11) P = 0.005**	**4.42 (2.01, 9.76) P<0.001**
Adjusted^c^	**1.00 (Referent)**	**0.98 (0.47, 2.05) P = 0.97**	**1.96 (1.21, 3.17) P = 0.006**	**3.58 (1.59, 8.06) P = 0.002**
Adjusted^d^	**1.00 (Referent)**	**0.94 (0.45, 1.99) P = 0.88**	**2.04 (1.26, 3.30) P = 0.004**	**4.36 (1.91, 9.93) P<0.001**

Note

a. Referent in each case is absence of malnutrition.

b. Model 1: Estimates adjusted for age, sex, ethnicity, Deyo-Charlson index, DRG type, and length of stay.

c. Model 2: Estimates adjusted for age, sex, ethnicity, Deyo-Charlson index, DRG type, length of stay and vascular procedure code category.

d. Model 3: Estimates adjusted for age, sex, ethnicity, DRG type, length of stay, vascular procedure code category, diabetes, hypertension, congestive heart failure, chronic obstructive pulmonary disease, cirrhosis, metastatic malignancy, and chronic kidney disease.

Next, the association of malnutrition status and mortality following hospital discharge was analysed separately for men and women. Nonspecific malnutrition and specific protein-calorie malnutrition groups were combined due to low power. The 90-day post-discharge mortality rates were 3.3% in women and 3.9% in men. The adjusted associations of 90-day post-discharge mortality in patients with nonspecific malnutrition or protein-energy malnutrition had similar directionality, effects sizes and significance in both men and women (Table 4). There is no significant effect modification of the nutrition status-mortality association on the basis of sex (interaction p = 0.30).

**Table 4:** Unadjusted and adjusted associations between nutrition status and 90-day post-discharge mortality relative to sex.

	Nutrition Status		
	No malnutrition^a^	At risk for malnutrition	Non-specific or Protein-energy malnutrition
Women (n = 2113)			
*90-day Post-Discharge Mortality*			
Crude	**1.00 (Referent)**	**1.69 (0.66, 4.32) P = 0.28**	**3.18 (1.75, 5.79) P<0.001**
Adjusted^b^	**1.00 (Referent)**	**1.40 (0.49, 4.01) P = 0.53**	**2.14 (1.08, 4.25) P = 0.029**
Men (n = 2319)			
*90-day Post-Discharge Mortality*			
Crude	**1.00 (Referent)**	**1.18 (0.42, 3.30) P = 0.31**	**3.37 (2.03, 5.59) P<0.001**
Adjusted^b^	**1.00 (Referent)**	**0.77 (0.26, 2.27) P = 0.63**	**2.36 (1.31, 4.26) P = 0.004**

Note

a. Referent in each case is absence of malnutrition.

b. Model 4: Estimates adjusted for age, ethnicity, Deyo-Charlson index, DRG type, length of stay and vascular procedure code category.

Finally, we analysed the association of malnutrition status with other outcomes following hospital discharge. Univariate data show that malnourished patients were significantly less likely to be discharged to home and more likely to be readmitted to hospital (Tables 2 & 5). Such significant associations were also present following multivariable adjustment, patients with malnutrition have a 52% lower odds of being discharged to home and a 50% higher odds of 30-day hospital readmission (Table 5).

**Table 5:** Unadjusted and adjusted associations between nutrition status and post-discharge outcomes (n = 4,432).

	Nutrition Status		
	No malnutrition^a^	At risk for malnutrition	Non-specific or Protein-energy malnutrition
*Discharge to Home*			
Crude	1.00 (Referent)	**0.22 (0.16, 0.29) P<0.001**	**0.17 (0.14, 0.22) P<0.001**
Adjusted^b^	1.00 (Referent)	**0.47 (0.33, 0.66) P<0.001**	**0.48 (0.36, 0.63) P<0.001**
*30-day Hospital Readmission*			
Crude	1.00 (Referent)	**1.87 (1.29, 2.69) P = 0.001**	**2.12 (1.62, 2.77) P<0.001**
Adjusted^c^	1.00 (Referent)	**1.44 (0.98, 2.11) P = 0.063**	**1.65 (1.24, 2.21) P = 0.001**

Note

a. Referent in each case is absence of malnutrition.

b. Model 5: Estimates adjusted for age, sex, ethnicity, Deyo-Charlson index, DRG type, length of stay and vascular procedure code category.

c. Model 6: Estimates adjusted for age, sex, ethnicity, Deyo-Charlson index, DRG type, length of stay and vascular procedure code category.

## Discussion

Malnutrition in hospitalized patients is a robust risk factor for adverse outcomes [45]. In our study we evaluated the association between malnutrition and major post-discharge outcomes in vascular surgery patients. We demonstrate that malnutrition in vascular surgery patients was strongly associated with out-of-hospital mortality. In addition to this, vascular surgery patients with malnutrition had a significantly less likelihood of being discharged to home compared to those without malnutrition. These findings confirm the intuitive reasoning that malnutrition contributes to adverse events in vascular surgery patients even after hospital discharge.

The ACC-AHA Guidelines for the Management of Patients with peripheral artery disease (PAD) recommend that patients should be treated with an interdisciplinary care team including a dietitian and should be prescribed a heart-healthy diet like all other cardiovascular disease patients [46]. Although this seems logical as PAD belongs within the spectrum cardiovascular diseases, the guideline also identifies the existence of an evidence gap in the role of dietary intervention to improve outcomes in PAD patients which is especially apparent peri-operatively. Also, in the Global Vascular guidelines on the management of critical limb-threatening ischemia, malnutrition is only mentioned to have an influence on wound healing and it is mentioned that a nutritionist should be included in a multidisciplinary team to prevent amputations in diabetic patients [47].

There is no specific evidence presented on nutritional diagnostics or interventions and consequently no recommendations are made in the ACC-AHA Guidelines. The current dietary evidence to prevent or treat vascular disease consists of retrospective and cross-sectional studies mainly with the outcome walking distance but lacks successful interventional clinical trials [48–52]. In the European guideline for abdominal aortic aneurysms it is recommended to measure serum albumin to assess the nutritional status of the patient [53]. This is based on research that identifies the association between pre-operative hypoalbuminemia and 30 day post-operative outcomes [18]. However, clinicians must recognize that albumin is a poor indicator of nutritional status since it is a negative acute phase protein and a low level may be more indicative of inflammation rather than malnutrition [54]. It is also advised to correct for any nutritional deficiencies by referral to an RD although the efficacy of this intervention has not been demonstrated by a randomized clinical trial [53]. Future research should focus more on dietary intervention for patients with vascular disease as a secondary prevention intervention but also as a peri-operative intervention to improve adverse outcomes and the effect of co-morbidities on malnutrition in vascular surgery patients.

The knowledge of how nutrition might influence post-discharge outcomes after vascular surgery can help surgeons to improve risk stratification and personalize postoperative care. If patients are electively admitted, prior dietary optimization could lower perioperative risk. Understanding what the risk of death comprises following vascular surgery is vital in the decision-making process for surgeon, patient and their family [55]. Unfortunately, no peri-operative risk scores include nutritional status for risk stratification in vascular surgery patients due to lack of evidence [56, 57]. Our data show that malnutrition is clearly related to the severity of co-morbidities, i.e. the Deyo-Charlson index, and therefore was included as a confounder in the multivariable analysis of outcomes. Taken into account the severity of co-morbidities malnutrition does show to be a robust risk factor for adverse outcomes following hospital discharge. The inclusion of the nutritional status in the pre-operative decision making process can aid in the counselling of patients and their family by providing information on prognostic factors.

Nutritional status is a potentially modifiable risk factor. In the postoperative setting, nutritional status optimization may help vascular surgery patients who are more likely to develop adverse events. This is collaborated by our findings that patients with malnutrition had a lower chance of discharge to home as well as higher mortality risk. These patients may benefit from an intensified follow-up in the care facility or in their own home. For example, in cardiac surgery patients who are discharged from the hospital, frequent home visits by a hospital nurse practitioner in the post-discharge period improved readmission and mortality rates [9, 58]. These kind of interventions aimed to improve post-discharge care could potentially improve outcomes for vascular surgery patients as well.

Potential limitations of this study include ascertainment bias as only vascular surgery patients deemed at nutritional risk were fully evaluated by an RD. This can limit the generalizability of the study findings. Despite multivariable adjustment, residual confounding is likely to be present. By determining covariates on the basis of ICD-9 codes it is likely that comorbidities are underestimated [59]. Though MGH and BWH contain 55% of hospital beds in Boston, we are not able to determine all readmissions to all hospitals for each patient. Lastly, although the BWH inpatient nutrition status evaluations have been shown to be predictive of outcomes, they are based on the malnutrition definitions guidelines that were recommended at the time of data collection and not perfectly aligned with the newest guidelines [5, 44]_ENREF_37.

The study has several strengths. The cohort contains a large number of vascular surgery patients which results in ample statistical power to detect a clinically relevant difference in 90-day post-discharge mortality if one exists. Linkage to the SSA Master Death file allows for out of hospital follow-up with high accuracy which was previously validated in our dataset [21]. The RPDR data sources have been validated for CPT code assignment [21], ICD-9 diagnosis [60, 61], and demographics [60]. Finally, all patients were screened for nutrition risk. Those at risk were assessed in-person for malnutrition risk by a highly trained nutrition professional rather than relying on self-reported malnutrition assessment surveys.

## Conclusions and implications

These data demonstrate that in vascular surgery patients, malnutrition is associated with increased post-discharge mortality. Further, those with malnutrition are more likely not to be discharged to home and to be readmitted to the hospital within 30 days. As malnutrition is a potentially modifiable risk factor, improvement of nutritional status may be a target for intervention in the already vulnerable vascular surgery patient population.

## Supporting information

S1 File Supplemental methods. (DOCX)

S2 File Supplemental data. (DOCX)

S1 Table Characteristics of vascular surgery procedure code categories stratified by 90-day post discharge mortality in the analytic cohort (n = 4432). (DOCX)

S2 Table Characteristics of vascular surgery procedure code categories stratified by nutritional status in the analytic cohort (n = 4432). (DOCX)

## References

[1] Corkins MR, Guenter P, DiMaria-Ghalili RA, et al. Malnutrition diagnoses in hospitalized patients: United States, 2010. *JPEN J Parenter Enteral Nutr*. 2014; 38: 186–95. doi: 10.1177/0148607113512154 24247093

[2] Correia MI and Waitzberg DL. The impact of malnutrition on morbidity, mortality, length of hospital stay and costs evaluated through a multivariate model analysis. *Clin Nutr*. 2003; 22: 235–9. doi: 10.1016/s0261-5614(02)00215-7 12765661

[3] Kondrup J, Johansen N, Plum LM, et al. Incidence of nutritional risk and causes of inadequate nutritional care in hospitals. *Clin Nutr*. 2002; 21: 461–8. doi: 10.1054/clnu.2002.0585 12468365

[4] McWhirter JP and Pennington CR. Incidence and recognition of malnutrition in hospital. *BMJ*. 1994; 308: 945–8. doi: 10.1136/bmj.308.6934.945 8173401 PMC2539799

[5] Mogensen KM, Robinson MK, Casey JD, et al. Nutritional Status and Mortality in the Critically Ill. *Crit Care Med*. 2015; 43: 2605–15. doi: 10.1097/CCM.0000000000001306 26427592

[6] Tewari N, Rodrigues J, Bothamley L, et al. The utilisation of the MUST nutritional screening tool on vascular surgical wards. *BMJ Qual Improv Rep*. 2013; 2. doi: 10.1136/bmjquality.u201374.w1122 26734198 PMC4652729

[7] WestergrenA, LindholmC, AxelssonC, et al. Prevalence of eating difficulties and malnutrition among persons within hospital care and special accommodations. *J Nutr Health Aging*. 2008; 12: 39–43. doi: 10.1007/BF02982162 18165843

[8] Boitano LT, Wang EC and Kibbe MR. Differential effect of nutritional status on vascular surgery outcomes in a Veterans Affairs versus private hospital setting. *Am J Surg*. 2012; 204: e27–37. doi: 10.1016/j.amjsurg.2012.07.023 23017254

[9] Havens JM, Columbus AB, Seshadri AJ, et al. Malnutrition at Intensive Care Unit Admission Predicts Mortality in Emergency General Surgery Patients. *JPEN J Parenter Enteral Nutr*. 2016: 148607116676592. 10.1177/0148607116676592 27821662

[10] Kassin MT, Owen RM, Perez SD, et al. Risk factors for 30-day hospital readmission among general surgery patients. *J Am Coll Surg*. 2012; 215: 322–30. doi: 10.1016/j.jamcollsurg.2012.05.024 22726893 PMC3423490

[11] Thomas MN, Kufeldt J, Kisser U, et al. Effects of malnutrition on complication rates, length ofhospital stay, and revenue in elective surgical patients in the G-DRG-system. *Nutrition*. 2016; 32: 249–54. doi: 10.1016/j.nut.2015.08.021 26688128

[12] Cederholm T, Jagren C and Hellstrom K. Outcome of protein-energy malnutrition in elderly medical patients. *Am J Med*. 1995; 98: 67–74. doi: 10.1016/S0002-9343(99)80082-5 7825621

[13] Norman K, Pichard C, Lochs H, et al. Prognostic impact of disease-related malnutrition. *Clin Nutr*. 2008; 27: 5–15. doi: 10.1016/j.clnu.2007.10.007 18061312

[14] O’Flynn J, Peake H, Hickson M, et al. The prevalence of malnutrition in hospitals can be reduced: results from three consecutive cross-sectional studies. *Clin Nutr*. 2005; 24: 1078–88. doi: 10.1016/j.clnu.2005.08.012 16219393

[15] Durkin MT, Mercer KG, McNulty MF, et al. Vascular surgical society of great britain and ireland: contribution of malnutrition to postoperative morbidity in vascular surgical patients. *Br J Surg*. 1999; 86: 702. doi: 10.1046/j.1365-2168.1999.0702a.x 10361208

[16] Stone PA, Flaherty SK, Aburahma AF, et al. Factors affecting perioperative mortality and wound-related complications following major lower extremity amputations. *Ann Vasc Surg*. 2006; 20: 209–16. doi: 10.1007/s10016-006-9009-z 16586027

[17] Hassen TA, Pearson S, Cowled PA, et al. Preoperative nutritional status predicts the severity of the systemic inflammatory response syndrome (SIRS) following major vascular surgery. *Eur J Vasc Endovasc Surg*. 2007; 33: 696–702. doi: 10.1016/j.ejvs.2006.12.006 17276097

[18] Inagaki E, Farber A, Eslami MH, et al. Preoperative hypoalbuminemia is associated with poor clinical outcomes after open and endovascular abdominal aortic aneurysm repair. *J Vasc Surg*. 2017; 66: 53–63 e1. doi: 10.1016/j.jvs.2016.10.110 28216349

[19] Murphy SN and Chueh HC. A security architecture for query tools used to access large biomedical databases. *Proc AMIA Symp*. 2002: 552–6. 12463885 PMC2244204

[20] Nalichowski R, Keogh D, Chueh HC, et al. Calculating the benefits of a Research Patient Data Repository. *AMIA Annu Symp Proc*. 2006: 1044. 17238663 PMC1839563

[21] Zager S, Mendu ML, Chang D, et al. Neighborhood poverty rate and mortality in patients receiving critical care in the academic medical center setting. *Chest*. 2011; 139: 1368–79. doi: 10.1378/chest.10-2594 21454401 PMC3109648

[22] Robinson MK, Mogensen KM, Casey JD, et al. The relationship among obesity, nutritional status, and mortality in the critically ill. *Crit Care Med*. 2015; 43: 87–100. doi: 10.1097/CCM.0000000000000602 25289931

[23] von Meijenfeldt GCI, van der Laan MJ, Zeebregts C, et al. Red cell distribution width at hospital discharge and out-of hospital outcomes in critically ill non-cardiac vascular surgery patients. *PLoS One*. 2018; 13: e0199654. doi: 10.1371/journal.pone.0199654 30183701 PMC6124728

[24] Fleisch A. [Basal metabolism standard and its determination with the "metabocalculator"]. *Helv Med Acta*. 1951; 18: 23–44. 14813607

[25] Jacobs DG, Jacobs DO, Kudsk KA, et al. Practice management guidelines for nutritional support of the trauma patient. *J Trauma*. 2004; 57: 660–78; discussion 79. doi: 10.1097/01.ta.0000135348.48525.a0 15454822

[26] McClave SA, Martindale RG, Vanek VW, et al. Guidelines for the Provision and Assessment of Nutrition Support Therapy in the Adult Critically Ill Patient: Society of Critical Care Medicine (SCCM) and American Society for Parenteral and Enteral Nutrition (A.S.P.E.N.). *JPEN J Parenter Enteral Nutr*. 2009; 33: 277–316. doi: 10.1177/0148607109335234 19398613

[27] Mogensen KM, Andrew BY, Corona JC, et al. Validation of the Society of Critical Care Medicine and American Society for Parenteral and Enteral Nutrition Recommendations for Caloric Provision to Critically Ill Obese Patients: A Pilot Study. *JPEN J Parenter Enteral Nutr*. 2016; 40: 713–21. doi: 10.1177/0148607115584001 25897016

[28] Barak N, Wall-Alonso E and Sitrin MD. Evaluation of stress factors and body weight adjustments currently used to estimate energy expenditure in hospitalized patients. *JPEN J Parenter Enteral Nutr*. 2002; 26: 231–8. doi: 10.1177/0148607102026004231 12090688

[29] Simopoulos AP. Obesity and body weight standards. *Annu Rev Public Health*. 1986; 7: 481–92. doi: 10.1146/annurev.pu.07.050186.002355 3487335

[30] Rapoport J, Gehlbach S, Lemeshow S, et al. Resource utilization among intensive care patients. Managed care vs traditional insurance. *Arch Intern Med*. 1992; 152: 2207–12. 1444680

[31] Charlson ME, Pompei P, Ales KL, et al. A new method of classifying prognostic comorbidity in longitudinal studies: development and validation. *J Chronic Dis*. 1987; 40: 373–83. doi: 10.1016/0021-9681(87)90171-8 3558716

[32] Liu V, Escobar GJ, Greene JD, et al. Hospital deaths in patients with sepsis from 2 independent cohorts. *JAMA*. 2014; 312: 90–2. doi: 10.1001/jama.2014.5804 24838355

[33] Quan H, Sundararajan V, Halfon P, et al. Coding algorithms for defining comorbidities in ICD-9-CM and ICD-10 administrative data. *Med Care*. 2005; 43: 1130–9. doi: 10.1097/01.mlr.0000182534.19832.83 16224307

[34] Elixhauser A, Steiner C and Palmer L. Clinical classifications software (CCS). In: HCUP HCaUP, (ed.). Rockville, MD: US Agency for Healthcare Research and Quality, 2014.

[35] *CPT 2004 Professional (Current Procedural Terminology (CPT) Professional)*. Chicago: American Medical Association, 2003.

[36] *CPT 2010 Standard Edition (CPT / Current Procedural Terminology)* Chicago: American Medical Association, 2009.

[37] Beebe M, Dalton JA, Espronceda M, et al. *CPT 2008 Standard Edition: Current Procedural Terminology (CPT / Current Procedural Terminology*). Chicago: American Medical Association, 2007.

[38] Boudreau AJ, Abraham M and Ahlman JT. *CPT 2012 Professional (Current Procedural Terminology (CPT) Professional)*. Chicago: American Medical Association, 2011.

[39] Horkan CM, Purtle SW, Mendu ML, et al. The association of acute kidney injury in the critically ill and postdischarge outcomes: a cohort study*. *Crit Care Med*. 2015; 43: 354–64. doi: 10.1097/CCM.0000000000000706 25474534

[40] Jencks SF, Williams MV and Coleman EA. Rehospitalizations among patients in the Medicare fee-for-service program. *N Engl J Med*. 2009; 360: 1418–28. doi: 10.1056/NEJMsa0803563 19339721

[41] Landrum L and Weinrich S. Readmission data for outcomes measurement: identifying and strengthening the empirical base. *Qual Manag Health Care*. 2006; 15: 83–95. doi: 10.1097/00019514-200604000-00003 16622357

[42] Purtle SW, Moromizato T, McKane CK, et al. The association of red cell distribution width at hospital discharge and out-of-hospital mortality following critical illness. *Crit Care Med*. 2014; 42: 918–29. doi: 10.1097/CCM.0000000000000118 24448196

[43] Goodwin AJ, Rice DA, Simpson KN, et al. Frequency, cost, and risk factors of readmissions among severe sepsis survivors. *Crit Care Med*. 2015; 43: 738–46. doi: 10.1097/CCM.0000000000000859 25746745 PMC4479267

[44] White JV, Guenter P, Jensen G, et al. Consensus statement: Academy of Nutrition and Dietetics and American Society for Parenteral and Enteral Nutrition: characteristics recommended for the identification and documentation of adult malnutrition (undernutrition). *JPEN J Parenter Enteral Nutr*. 2012; 36: 275–83. doi: 10.1177/0148607112440285 22535923

[45] Felder S, Lechtenboehmer C, Bally M, et al. Association of nutritional risk and adverse medical outcomes across different medical inpatient populations. *Nutrition*. 2015; 31: 1385–93. doi: 10.1016/j.nut.2015.06.007 26429660

[46] Gerhard-Herman MD, Gornik HL, Barrett C, et al. 2016 AHA/ACC Guideline on the Management of Patients With Lower Extremity Peripheral Artery Disease: A Report of the American College of Cardiology/American Heart Association Task Force on Clinical Practice Guidelines. *Circulation*. 2017; 135: e726–e79. doi: 10.1161/CIR.0000000000000471 27840333 PMC5477786

[47] Conte MS, Bradbury AW, Kolh P, et al. Global Vascular Guidelines on the Management of Chronic Limb-Threatening Ischemia. *Eur J Vasc Endovasc Surg*. 2019; 58: S1–S109 e33. doi: 10.1016/j.ejvs.2019.05.006 31182334 PMC8369495

[48] Brostow DP, Hirsch AT, Collins TC, et al. The role of nutrition and body composition in peripheral arterial disease. *Nat Rev Cardiol*. 2012; 9: 634–43. doi: 10.1038/nrcardio.2012.117 22922595 PMC4535926

[49] Brostow DP, Hirsch AT, Pereira MA, et al. Nutritional status and body composition in patients with peripheral arterial disease: A cross-sectional examination of disease severity and quality of life. *Ecol Food Nutr*. 2016; 55: 87–109. doi: 10.1080/03670244.2015.1072817 26654593 PMC6456054

[50] McDermott MM, Liu K, Ferrucci L, et al. Vitamin D status, functional decline, and mortality in peripheral artery disease. *Vasc Med*. 2014; 19: 18–26. doi: 10.1177/1358863X13518364 24442622 PMC5139679

[51] McDermott MM, Mehta S, Ahn H, et al. Atherosclerotic risk factors are less intensively treated in patients with peripheral arterial disease than in patients with coronary artery disease. *J Gen Intern Med*. 1997; 12: 209–15. doi: 10.1046/j.1525-1497.1997.012004209.x 9127224 PMC1497093

[52] Nosova EV, Conte MS and Grenon SM. Advancing beyond the "heart-healthy diet" for peripheral arterial disease. *J Vasc Surg*. 2015; 61: 265–74. doi: 10.1016/j.jvs.2014.10.022 25534981 PMC4275620

[53] Wanhainen A, Verzini F, Van Herzeele I, et al. Editor’s Choice—European Society for Vascular Surgery (ESVS) 2019 Clinical Practice Guidelines on the Management of Abdominal Aorto-iliac Artery Aneurysms. *Eur J Vasc Endovasc Surg*. 2019; 57: 8–93. doi: 10.1016/j.ejvs.2018.09.020 30528142

[54] Jensen GL, Bistrian B, Roubenoff R, et al. Malnutrition syndromes: a conundrum vs continuum. *JPEN J Parenter Enteral Nutr*. 2009; 33: 710–6. doi: 10.1177/0148607109344724 19892905

[55] Schenker Y, Fernandez A, Sudore R, et al. Interventions to improve patient comprehension in informed consent for medical and surgical procedures: a systematic review. *Med Decis Making*. 2011; 31: 151–73. doi: 10.1177/0272989X10364247 20357225 PMC5419590

[56] Kolh P, De Hert S and De Rango P. The Concept of Risk Assessment and Being Unfit for Surgery. *Eur J Vasc Endovasc Surg*. 2016; 51: 857–66. doi: 10.1016/j.ejvs.2016.02.004 27053098

[57] Von Meijenfeldt GC, Van Der Laan MJ, Zeebregts CJ, et al. Risk assessment and risk scores in the management of aortic aneurysms. *J Cardiovasc Surg (Torino)*. 2016; 57: 162–71. 26698033

[58] Hall MH, Esposito RA, Pekmezaris R, et al. Cardiac surgery nurse practitioner home visits prevent coronary artery bypass graft readmissions. *Ann Thorac Surg*. 2014; 97: 1488–93; discussion 93–5. doi: 10.1016/j.athoracsur.2013.12.049 24612701

[59] Linde-Zwirble WT and Angus DC. Severe sepsis epidemiology: sampling, selection, and society. *Crit Care*. 2004; 8: 222–6. doi: 10.1186/cc2917 15312201 PMC522859

[60] Hug BL, Lipsitz SR, Seger DL, et al. Mortality and drug exposure in a 5-year cohort of patients with chronic liver disease. *Swiss Med Wkly*. 2009; 139: 737–46. 19924579 10.4414/smw.2009.12686

[61] Moromizato T, Litonjua AA, Braun AB, et al. Association of low serum 25-hydroxyvitamin D levels and sepsis in the critically ill. *Crit Care Med*. 2014; 42: 97–107. doi: 10.1097/CCM.0b013e31829eb7af 23982028

