# Supplementary material for: Nutritional status and out-of-hospital mortality in vascular surgery patients
Source: PLoS One. 2022 Jul 21;17(7):e0270396. doi: 10.1371/journal.pone.0270396 (PMC9302752; doi:10.1371/journal.pone.0270396)
Supplement: S1 File — (DOCX) [file pone.0270396.s001.docx]

**Supplemental Methods**

**Malnutrition Criteria**

**Severe Protein-Energy Malnutrition**

Three of the following criteria must be met but not all criteria are required:

Significant, disease-related, weight loss of > 15% of usual weight within the past six months

< 70% ideal body weight

Serum albumin < 2.1 g/dL

TLC ≤ 800 mm^3^ **

Transferrin < 100 mg/dL*** (calculated: total iron binding capacity/1.45)

Overt signs of muscle wasting on physical exam

Inadequate kcal intake (< 50% of estimated needs for 3 days or < 75% of estimated needs for 7 days)

**Moderate Protein-Energy Malnutrition**

Two of the following criteria must be met but not all criteria are required:

Significant, disease-related, weight loss of 10%-15% of usual weight within the past six months

70%-84% of ideal body weight

Serum albumin 2.1 g/dL-2.7 g/dL*

TLC 800-1199 mm^3^ **

Transferrin 100-149 mg/dL***

**Mild Protein-Energy Malnutrition**

Two of the following criteria must be met but not all criteria are required:

Significant, disease-related, weight loss of 5%-9% of usual weight within the past six months

85%-94% of ideal body weight

Serum albumin 2.8-3.4 g/dL

TLC 1200-1499 mm^3^ **

Transferrin 150-199 mg/dL***

**Malnutrition Criteria**

**Non-Specific Protein-Energy Malnutrition**

The patient has known nutritional risk with metabolic stress and/or overt signs of malnutrition without supporting anthropometric or biochemical data. Clinical judgment is required to make this classification.

Examples include:

Inadequate nutrient intake of kcal, protein, and micronutrients (< 50% of estimated needs for three days or < 75% of estimated needs for 7 days) concurrent with significant stress or injury; examples include trauma, major burn, short-bowel syndrome, severe pancreatitis, enterocutaneous fistula, inflammatory bowel disease

Overt wasting of muscle and fat stores without supporting clinical measures (e.g, anthropometric data unavailable, weight history unavailable, unreliable admission albumin)

Single nutrient deficiency

Multiple nutrient deficiencies

Note: Total lymphocyte count (TLC) = [%lymphocytes x WBC (mm^3^)]/100

*Only admission serum albumin is used because of difficulties in interpretation after hydration; additionally, serum albumin is not used if the patient is transferred after a prolonged stay at an outside hospital. If the admission albumin from the outside hospital is available, that may be used for evaluation.

**Excludes patients currently undergoing chemotherapy or radiation, or one month post-chemotherapy or radiation; currently receiving high-dose steroid therapy defined as ≥ 60 mg/day of prednisone or equivalent; suffering from leukemia; current infection

***Transferrin may only be used in an iron-replete patient who has not received blood transfusions.

**Energy Needs Determination**

Energy needs are determined by the following: for BMI < 30 kg/m^2^, basal metabolic rate is calculated based on body surface area (1) and age, then activity and stress factors are applied based on severity of illness (patients are generally fed between 30-35 kcal/kg). For BMI 30-35 kg/m^2^ we use adjusted weight for obesity (2), calculate resting metabolic rate, apply activity factor and metabolic stress factor based on severity of illness (3) (patients are generally fed between 25-35 kcal/kg). Patients with BMI 35-50 kg/m^2^ are fed at 14 kcal/kg dry weight, and those with BMI > 50 kg/m^2^ are fed at 25 kcal/kg ideal body weight (4, 5).

**Vascular Surgery CPT Codes**

Current Procedural Terminology (CPT) codes were assigned to the vascular surgery procedure by the procedure operator during daily billing. Details of the CPT codes utilized in vascular surgery practice and in our study are presented below. The CPT code set maintained and published yearly by the American Medical Association (6-9).

Endovascular

34813, 34820, 34825, 34826, 37195, 37201, 37202, 37203, 37204, 35471, 34800, 34802, 34803, 34804, 34805, 34808, 34900, 37205, 37206, 37207, 37208, 35452, 35454, 35456, 35458, 35459, 35470, 35472, 35473, 35474, 35475, 35493.

Arterial bypass

34813, 35556, 35565, 35566, 35571, 35606, 35621, 35623, 35626, 35631, 35646, 35647, 35654, 35656, 35661, 35663, 35665, 35666, 35671, 35546, 35641, 35681, 35682, 35583, 35585.

Artery Exposure / Exploration

34820, 34812, 34833, 35701, 35721, 35761, 35800, 35840, 35860, 35741.

Arteriovenous fistula

36821, 36825, 36832.

Decompression

27600, 27601, 27602, 27892, 27894, 24495, 25020, 25023.

Embolectomy Or Thrombectomy

34101, 35875, 35876, 34001, 34151, 34201, 34203, 34111, 36831, 36870, 34401, 34451.

Endovascular other

34825, 34826, 37195, 37201, 37202, 37203, 37204, Graft Excision, 35903, 35907.

Major amputation

27290, 27590, 27592, 27594, 27596, 27880, 27884, 27886, 28800, 27882, 27295, 27598, 24900, 24920, 24930, 25900, 25905, 25907, 25909.

Minor amputation

28805, 28810, 28820, 28825, 26910, 26951, 26952.

Repair Blood Vessel

35471, 35190, 35201, 35206, 35207, 35221, 35226, 35231, 35236, 35251, 35256, 35266, 35281, 35286, 35301, 35879, 36833, 34502.

Repair of aneurysm or occlusive disease

34800, 34802, 34803, 34804, 34805, 34808, 34900, 35001, 35011, 35081, 35091, 35102, 35111, 35121, 35141, 35151, 34830, 35002, 35082, 35092, 35103, 35142, 35045.

Stent placement

37205, 37206, 37207, 37208.

Thromboendarterectomy

35321, 35331, 35341, 35351, 35355, 35371, 35372, 35381.

Transluminal Balloon Angioplasty

35452, 35454, 35456, 35458, 35459, 35470, 35472, 35473, 35474, 35475, 35476.

Transluminal Peripheral Atherectomy 35493.

Vessel ligation 37607, 37609, 37617, 37618.

**Planned Readmission and Diagnostic Related Groups codes**

DRG codes that are commonly associated with planned readmissions in addition to DRGs for transplantation, procedures related to pregnancy, and psychiatric issues (10, 11). To evaluate unplanned readmissions, we excluded readmissions with DRG codes that are commonly associated with planned readmissions: DRG 001, 075, 105, 109, 110, 113, 120, 209, 263, 315, 336, 410, 462, 478, 515, 517, 518, 527, and 533 (11) in addition to DRGs for transplantation: 103, 302, 480, 481 and 495; procedures related to pregnancy: 364, 370, 371, 372, 374, 381; dental procedures: 185, 187; and psychiatric issues: 425, 426, 428, 430, 433, 434, 435, 521, and 523. Seventy three 30-day readmissions were classified as planned readmissions and not included as 30-day readmissions in Tables 1, 2 and 5.

**References**

1. Fleisch A. [Basal metabolism standard and its determination with the "metabocalculator"]. Helvetica medica acta. 1951;18(1):23-44.

2. Karkeck J. Adjusted body weight for obesity. American Dietetic Association Renal Dietitians Practice Group Newsletter. 1984;3:6.

3. Barak N, Wall-Alonso E, Sitrin MD. Evaluation of stress factors and body weight adjustments currently used to estimate energy expenditure in hospitalized patients. JPEN J Parenter Enteral Nutr. 2002;26(4):231-8.

4. Jacobs DG, Jacobs DO, Kudsk KA, Moore FA, Oswanski MF, Poole GV, et al. Practice management guidelines for nutritional support of the trauma patient. J Trauma. 2004;57(3):660-78; discussion 79.

5. McClave SA, Martindale RG, Vanek VW, McCarthy M, Roberts P, Taylor B, et al. Guidelines for the Provision and Assessment of Nutrition Support Therapy in the Adult Critically Ill Patient: Society of Critical Care Medicine (SCCM) and American Society for Parenteral and Enteral Nutrition (A.S.P.E.N.). JPEN J Parenter Enteral Nutr. 2009;33(3):277-316.

6. CPT 2004 Professional (Current Procedural Terminology (CPT) Professional). Chicago: American Medical Association; 2003.

7. CPT 2010 Standard Edition (CPT / Current Procedural Terminology) Chicago: American Medical Association; 2009.

8. Beebe M, Dalton JA, Espronceda M, Evans DD, Glenn RL. CPT 2008 Standard Edition: Current Procedural Terminology (CPT / Current Procedural Terminology). Chicago: American Medical Association; 2007.

9. Boudreau AJ, Abraham M, Ahlman JT. CPT 2012 Professional (Current Procedural Terminology (CPT) Professional). Chicago: American Medical Association; 2011.

10. Horkan CM, Purtle SW, Mendu ML, Moromizato T, Gibbons FK, Christopher KB. The association of acute kidney injury in the critically ill and postdischarge outcomes: a cohort study*. Critical care medicine. 2015;43(2):354-64.

11. Jencks SF, Williams MV, Coleman EA. Rehospitalizations among patients in the Medicare fee-for-service program. N Engl J Med. 2009;360(14):1418-28.
