# Supplementary material for: Nutritional status and out-of-hospital mortality in vascular surgery patients
Source: PLoS One. 2022 Jul 21;17(7):e0270396. doi: 10.1371/journal.pone.0270396 (PMC9302752; doi:10.1371/journal.pone.0270396)
Supplement: S2 File — (DOCX) [file pone.0270396.s002.docx]

**Supplemental data file**

CPT codes and description

| **CPT code** | **CPT and Description** | **Vascular Surgery Procedure Code Categories** | **Vascular Surgery Detail** |
| --- | --- | --- | --- |
| 34813 | 34813 Placement of femoral-femoral prosthetic graft during endovascular aortic aneurysm repair (List separately in addition to code for primary procedure) | Arterial bypass | Endovascular |
| 35556 | 35556 Bypass graft, with vein; femoral-popliteal | Arterial bypass | Non-Endovascular |
| 35565 | 35565 Bypass graft, with vein; iliofemoral | Arterial bypass | Non-Endovascular |
| 35566 | 35566 Bypass graft, with vein; femoral-anterior tibial, posterior tibial, peroneal artery or other distal vessels | Arterial bypass | Non-Endovascular |
| 35571 | 35571 Bypass graft, with vein; popliteal-tibial, -peroneal artery or other distal vessels | Arterial bypass | Non-Endovascular |
| 35606 | 35606 Bypass graft, with other than vein; carotid-subclavian | Arterial bypass | Non-Endovascular |
| 35621 | 35621 Bypass graft, with other than vein; axillary-femoral | Arterial bypass | Non-Endovascular |
| 35623 | 35623 Bypass graft, with other than vein; axillary-popliteal or -tibial | Arterial bypass | Non-Endovascular |
| 35626 | 35626 Bypass graft, with other than vein; aortosubclavian or carotid | Arterial bypass | Non-Endovascular |
| 35631 | 35631 Bypass graft, with other than vein; aortoceliac, aortomesenteric, aortorenal | Arterial bypass | Non-Endovascular |
| 35646 | 35646 Bypass graft, with other than vein; aortobifemoral | Arterial bypass | Non-Endovascular |
| 35647 | 35647 Bypass graft, with other than vein; aortofemoral | Arterial bypass | Non-Endovascular |
| 35654 | 35654 Bypass graft, with other than vein; axillary-femoral-femoral | Arterial bypass | Non-Endovascular |
| 35656 | 35656 Bypass graft, with other than vein; femoral-popliteal | Arterial bypass | Non-Endovascular |
| 35661 | 35661 Bypass graft, with other than vein; femoral-femoral | Arterial bypass | Non-Endovascular |
| 35663 | 35663 Bypass graft, with other than vein; ilioiliac | Arterial bypass | Non-Endovascular |
| 35665 | 35665 Bypass graft, with other than vein; iliofemoral | Arterial bypass | Non-Endovascular |
| 35666 | 35666 Bypass graft, with other than vein; femoral-anterior tibial, posterior tibial, or peroneal artery | Arterial bypass | Non-Endovascular |
| 35671 | 35671 Bypass graft, with other than vein; popliteal-tibial or -peroneal artery | Arterial bypass | Non-Endovascular |
| 35546 | 35546 Bypass graft, with vein; aortofemoral or bifemoral | Arterial bypass | Non-Endovascular |
| 35641 | 35641 Bypass graft, with other than vein; aortoiliac or bi-iliac | Arterial bypass | Non-Endovascular |
| 35681 | 35681 Bypass graft; composite, prosthetic and vein (List separately in addition to code for primary procedure) | Arterial bypass | Non-Endovascular |
| 35682 | 35682 Bypass graft; autogenous composite, two segments of veins from two locations (List separately in addition to code for primary procedure) | Arterial bypass | Non-Endovascular |
| 35583 | 35583 In-situ vein bypass; femoral-popliteal | Arterial bypass | Non-Endovascular |
| 35585 | 35585 In-situ vein bypass; femoral-anterior tibial, posterior tibial, or peroneal artery | Arterial bypass | Non-Endovascular |
| 34820 | 34820 Open iliac artery exposure for delivery of endovascular prosthesis or iliac occlusion during endovascular therapy, by abdominal or retroperitoneal incision, unilateral | Artery Exposure / Exploration | Endovascular |
| 34812 | 34812 Open femoral artery exposure for delivery of endovascular prosthesis, by groin incision, unilateral | Artery Exposure / Exploration | Non-Endovascular |
| 34833 | 34833 Open iliac artery exposure with creation of conduit for delivery of infrarenal aortic or iliac endovascular prosthesis, by abdominal or retroperitoneal incision, unilateral | Artery Exposure / Exploration | Non-Endovascular |
| 35701 | 35701 Exploration (not followed by surgical repair), with or without lysis of artery; carotid artery | Artery Exposure / Exploration | Non-Endovascular |
| 35721 | 35721 Exploration (not followed by surgical repair), with or without lysis of artery; femoral artery | Artery Exposure / Exploration | Non-Endovascular |
| 35761 | 35761 Exploration (not followed by surgical repair), with or without lysis of artery; other vessels | Artery Exposure / Exploration | Non-Endovascular |
| 35800 | 35800 Exploration for postoperative hemorrhage, thrombosis or infection; neck | Artery Exposure / Exploration | Non-Endovascular |
| 35840 | 35840 Exploration for postoperative hemorrhage, thrombosis or infection; abdomen | Artery Exposure / Exploration | Non-Endovascular |
| 35860 | 35860 Exploration for postoperative hemorrhage, thrombosis or infection; extremity | Artery Exposure / Exploration | Non-Endovascular |
| 35741 | 35741 Exploration (not followed by surgical repair), with or without lysis of artery; popliteal artery | Artery Exposure / Exploration | Non-Endovascular |
| 36821 | 36821 Arteriovenous anastomosis, open; direct, any site (eg, Cimino type) (separate procedure) | AVF | Non-Endovascular |
| 36825 | 36825 Creation of arteriovenous fistula by other than direct arteriovenous anastomosis (separate procedure); autogenous graft | AVF | Non-Endovascular |
| 36832 | 36832 Revision, open, arteriovenous fistula; without thrombectomy, autogenous or nonautogenous dialysis graft (separate procedure) | AVF | Non-Endovascular |
| 27600 | 27600 Decompression fasciotomy, leg; anterior and/or lateral compartments only | Decompression | Non-Endovascular |
| 27601 | 27601 Decompression fasciotomy, leg; posterior compartment(s) only | Decompression | Non-Endovascular |
| 27602 | 27602 Decompression fasciotomy, leg; anterior and/or lateral, and posterior compartment(s) | Decompression | Non-Endovascular |
| 27892 | 27892 Decompression fasciotomy, leg; anterior and/or lateral compartments only, with debridement of nonviable muscle and/or nerve | Decompression | Non-Endovascular |
| 27894 | 27894 Decompression fasciotomy, leg; anterior and/or lateral, and posterior compartment(s), with debridement of nonviable muscle and/or nerve | Decompression | Non-Endovascular |
| 24495 | 24495 Decompression fasciotomy, forearm, with brachial artery exploration | Decompression | Non-Endovascular |
| 25020 | 25020 Decompression fasciotomy, forearm and/or wrist, flexor OR extensor compartment; without debridement of nonviable muscle and/or nerve | Decompression | Non-Endovascular |
| 25023 | 25023 Decompression fasciotomy, forearm and/or wrist, flexor OR extensor compartment; with debridement of nonviable muscle and/or nerve | Decompression | Non-Endovascular |
| 34101 | 34101 Embolectomy or thrombectomy, with or without catheter; axillary, brachial, innominate, subclavian artery, by arm incision 34111 Embolectomy or thrombectomy, with or without catheter; radial or ulnar artery, by arm incision | Embolectomy Or Thrombectomy | Non-Endovascular |
| 35875 | 35875 Thrombectomy of arterial or venous graft (other than hemodialysis graft or fistula); | Embolectomy Or Thrombectomy | Non-Endovascular |
| 35876 | 35876 Thrombectomy of arterial or venous graft (other than hemodialysis graft or fistula); with revision of arterial or venous graft | Embolectomy Or Thrombectomy | Non-Endovascular |
| 34001 | 34001 Embolectomy or thrombectomy, with or without catheter; carotid, subclavian or innominate artery, by neck incision | Embolectomy Or Thrombectomy | Non-Endovascular |
| 34151 | 34151 Embolectomy or thrombectomy, with or without catheter; renal, celiac, mesentery, aortoiliac artery, by abdominal incision | Embolectomy Or Thrombectomy | Non-Endovascular |
| 34201 | 34201 Embolectomy or thrombectomy, with or without catheter; femoropopliteal, aortoiliac artery, by leg incision | Embolectomy Or Thrombectomy | Non-Endovascular |
| 34203 | 34203 Embolectomy or thrombectomy, with or without catheter; popliteal-tibio-peroneal artery, by leg incision | Embolectomy Or Thrombectomy | Non-Endovascular |
| 34111 | 34111 Embolectomy or thrombectomy, with or without catheter | Embolectomy Or Thrombectomy | Non-Endovascular |
| 36831 | 36831 Thrombectomy, open, arteriovenous fistula without revision, autogenous or nonautogenous dialysis graft (separate procedure) | Embolectomy Or Thrombectomy | Non-Endovascular |
| 36870 | 36870 Thrombectomy, percutaneous, arteriovenous fistula, autogenous or nonautogenous graft (includes mechanical thrombus extraction and intra-graft thrombolysis) | Embolectomy Or Thrombectomy | Non-Endovascular |
| 34401 | 34401 Thrombectomy, direct or with catheter; vena cava, iliac vein, by abdominal incision | Embolectomy Or Thrombectomy | Non-Endovascular |
| 34451 | 34451 Thrombectomy, direct or with catheter; vena cava, iliac, femoropopliteal vein, by abdominal and leg incision | Embolectomy Or Thrombectomy | Non-Endovascular |
| 34825 | 34825 Placement of proximal or distal extension prosthesis for endovascular repair of infrarenal abdominal aortic or iliac aneurysm, false aneurysm, or dissection; initial vessel | Endovascular other | Endovascular |
| 34826 | 34826 Placement of proximal or distal extension prosthesis for endovascular repair of infrarenal abdominal aortic or iliac aneurysm, false aneurysm, or dissection; each additional vessel (List separately in addition to code for primary procedure) | Endovascular other | Endovascular |
| 37195 | 37195 Thrombolysis, cerebral, by intravenous infusion | Endovascular other | Endovascular |
| 37201 | 37201 Transcatheter therapy, infusion for thrombolysis other than coronary | Endovascular other | Endovascular |
| 37202 | 37202 Transcatheter therapy, infusion other than for thrombolysis, any type (eg, spasmolytic, vasoconstrictive) | Endovascular other | Endovascular |
| 37203 | 37203 Transcatheter retrieval, percutaneous, of intravascular foreign body (eg, fractured venous or arterial catheter) | Endovascular other | Endovascular |
| 37204 | 37204 Transcatheter occlusion or embolization (eg, for tumor destruction, to achieve hemostasis, to occlude a vascular malformation), percutaneous, any method, non-central nervous system, non-head or neck | Endovascular other | Endovascular |
| 35903 | 35903 Excision of infected graft; extremity | Graft Excision | Non-Endovascular |
| 35907 | 35907 Excision of infected graft; abdomen | Graft Excision | Non-Endovascular |
| 27290 | 27290 Interpelviabdominal amputation (hindquarter amputation) | Major amputation | Non-Endovascular |
| 27590 | 27590 Amputation, thigh, through femur, any level; | Major amputation | Non-Endovascular |
| 27592 | 27592 Amputation, thigh, through femur, any level; open, circular (guillotine) | Major amputation | Non-Endovascular |
| 27594 | 27594 Amputation, thigh, through femur, any level; secondary closure or scar revision | Major amputation | Non-Endovascular |
| 27596 | 27596 Amputation, thigh, through femur, any level; re-amputation | Major amputation | Non-Endovascular |
| 27880 | 27880 Amputation, leg, through tibia and fibula; | Major amputation | Non-Endovascular |
| 27884 | 27884 Amputation, leg, through tibia and fibula; secondary closure or scar revision | Major amputation | Non-Endovascular |
| 27886 | 27886 Amputation, leg, through tibia and fibula; re-amputation | Major amputation | Non-Endovascular |
| 28800 | 28800 Amputation, foot; midtarsal (eg, Chopart type procedure) | Major amputation | Non-Endovascular |
| 27882 | 27882 Amputation Procedures on the Leg (Tibia and Fibula) and Ankle Joint | Major amputation | Non-Endovascular |
| 27295 | 27295 Disarticulation of hip | Major amputation | Non-Endovascular |
| 27598 | 27598 Disarticulation at knee | Major amputation | Non-Endovascular |
| 24900 | 24900 Amputation, arm through humerus; with primary closure | Major amputation | Non-Endovascular |
| 24920 | 24920 Amputation, arm through humerus; open, circular (guillotine) | Major amputation | Non-Endovascular |
| 24930 | 24930 Amputation, arm through humerus; re-amputation | Major amputation | Non-Endovascular |
| 25900 | 25900 Amputation, forearm, through radius and ulna; | Major amputation | Non-Endovascular |
| 25905 | 25905 Amputation, forearm, through radius and ulna; open, circular (guillotine) | Major amputation | Non-Endovascular |
| 25907 | 25907 Amputation, forearm, through radius and ulna; secondary closure or scar revision | Major amputation | Non-Endovascular |
| 25909 | 25909 Amputation, forearm, through radius and ulna; re-amputation | Major amputation | Non-Endovascular |
| 28805 | 28805 Amputation, foot; transmetatarsal | Minor amputation | Non-Endovascular |
| 28810 | 28810 Amputation, metatarsal, with toe, single | Minor amputation | Non-Endovascular |
| 28820 | 28820 Amputation, toe; metatarsophalangeal joint | Minor amputation | Non-Endovascular |
| 28825 | 28825 Amputation, toe; interphalangeal joint | Minor amputation | Non-Endovascular |
| 26910 | 26910 Amputation, metacarpal, with finger or thumb (ray amputation), single, with or without interosseous transfer | Minor amputation | Non-Endovascular |
| 26951 | 26951 Amputation, finger or thumb, primary or secondary, any joint or phalanx, single, including neurectomies; with direct closure | Minor amputation | Non-Endovascular |
| 26952 | 26952 Amputation, finger or thumb, primary or secondary, any joint or phalanx, single, including neurectomies; with local advancement flaps (V-Y, hood) | Minor amputation | Non-Endovascular |
| 35471 | 35471 Transluminal balloon angioplasty, percutaneous; renal or visceral artery | Repair Blood Vessel | Endovascular |
| 35190 | 35190 Repair, acquired or traumatic arteriovenous fistula; extremities | Repair Blood Vessel | Non-Endovascular |
| 35201 | 35201 Repair blood vessel, direct; neck | Repair Blood Vessel | Non-Endovascular |
| 35206 | 35206 Repair blood vessel, direct; upper extremity | Repair Blood Vessel | Non-Endovascular |
| 35207 | 35207 Repair blood vessel, direct; hand, finger | Repair Blood Vessel | Non-Endovascular |
| 35221 | 35221 Repair blood vessel, direct; intra-abdominal | Repair Blood Vessel | Non-Endovascular |
| 35226 | 35226 Repair blood vessel, direct; lower extremity | Repair Blood Vessel | Non-Endovascular |
| 35231 | 35231 Repair blood vessel with vein graft; neck | Repair Blood Vessel | Non-Endovascular |
| 35236 | 35236 Repair blood vessel with vein graft; upper extremity | Repair Blood Vessel | Non-Endovascular |
| 35251 | 35251 Repair blood vessel with vein graft; intra-abdominal | Repair Blood Vessel | Non-Endovascular |
| 35256 | 35256 Repair blood vessel with vein graft; lower extremity | Repair Blood Vessel | Non-Endovascular |
| 35266 | 35266 Repair blood vessel with graft other than vein; upper extremity | Repair Blood Vessel | Non-Endovascular |
| 35281 | 35281 Repair blood vessel with graft other than vein; intra-abdominal | Repair Blood Vessel | Non-Endovascular |
| 35286 | 35286 Repair blood vessel with graft other than vein; lower extremity | Repair Blood Vessel | Non-Endovascular |
| 35301 | 35301 Thromboendarterectomy, with or without patch graft; carotid, vertebral, subclavian, by neck incision | Repair Blood Vessel | Non-Endovascular |
| 35879 | 35879 Revision, lower extremity arterial bypass, without thrombectomy, open; with vein patch angioplasty | Repair Blood Vessel | Non-Endovascular |
| 36833 | 36833 Revision, open, arteriovenous fistula; with thrombectomy, autogenous or nonautogenous dialysis graft (separate procedure) | Repair Blood Vessel | Non-Endovascular |
| 34502 | 34502 Reconstruction of vena cava, any method | Repair Blood Vessel | Non-Endovascular |
| 34800 | 34800 Endovascular repair of infrarenal abdominal aortic aneurysm or dissection; using aorto-aortic tube prosthesis | Repair of aneurysm or occlusive disease | Endovascular |
| 34802 | 34802 Endovascular repair of infrarenal abdominal aortic aneurysm or dissection; using modular bifurcated prosthesis (one docking limb) | Repair of aneurysm or occlusive disease | Endovascular |
| 34803 | 34803 Endovascular repair of infrarenal abdominal aortic aneurysm or dissection; using modular bifurcated prosthesis (two docking limbs) | Repair of aneurysm or occlusive disease | Endovascular |
| 34804 | 34804 Endovascular repair of infrarenal abdominal aortic aneurysm or dissection; using unibody bifurcated prosthesis | Repair of aneurysm or occlusive disease | Endovascular |
| 34805 | 34805 Endovascular repair of infrarenal abdominal aortic aneurysm or dissection; using aorto-uniiliac or aorto-unifemoral prosthesis | Repair of aneurysm or occlusive disease | Endovascular |
| 34808 | 34808 Endovascular placement of iliac artery occlusion device (List separately in addition to code for primary procedure) | Repair of aneurysm or occlusive disease | Endovascular |
| 34900 | 34900 Endovascular graft placement for repair of iliac artery (eg, aneurysm, pseudoaneurysm, arteriovenous malformation, trauma) | Repair of aneurysm or occlusive disease | Endovascular |
| 35001 | 35001 Direct repair of aneurysm, pseudoaneurysm, or excision (partial or total) and graft insertion, with or without patch graft; for aneurysm and associated occlusive disease, carotid, subclavian artery, by neck incision | Repair of aneurysm or occlusive disease | Non-Endovascular |
| 35011 | 35011 Direct repair of aneurysm, pseudoaneurysm, or excision (partial or total) and graft insertion, with or without patch graft; for aneurysm and associated occlusive disease, axillary-brachial artery, by arm incision | Repair of aneurysm or occlusive disease | Non-Endovascular |
| 35081 | 35081 Direct repair of aneurysm, pseudoaneurysm, or excision (partial or total) and graft insertion, with or without patch graft; for aneurysm, pseudoaneurysm, and associated occlusive disease, abdominal aorta | Repair of aneurysm or occlusive disease | Non-Endovascular |
| 35091 | 35091 Direct repair of aneurysm, pseudoaneurysm, or excision (partial or total) and graft insertion, with or without patch graft; for aneurysm, pseudoaneurysm, and associated occlusive disease, abdominal aorta involving visceral vessels (mesenteric, celiac, renal) | Repair of aneurysm or occlusive disease | Non-Endovascular |
| 35102 | 35102 Direct repair of aneurysm, pseudoaneurysm, or excision (partial or total) and graft insertion, with or without patch graft; for aneurysm, pseudoaneurysm, and associated occlusive disease, abdominal aorta involving iliac vessels (common, hypogastric, external) | Repair of aneurysm or occlusive disease | Non-Endovascular |
| 35111 | 35111 Direct repair of aneurysm, pseudoaneurysm, or excision (partial or total) and graft insertion, with or without patch graft; for aneurysm, pseudoaneurysm, and associated occlusive disease, splenic artery | Repair of aneurysm or occlusive disease | Non-Endovascular |
| 35121 | 35121 Direct repair of aneurysm, pseudoaneurysm, or excision (partial or total) and graft insertion, with or without patch graft; for aneurysm, pseudoaneurysm, and associated occlusive disease, hepatic, celiac, renal, or mesenteric artery | Repair of aneurysm or occlusive disease | Non-Endovascular |
| 35141 | 35141 Direct repair of aneurysm, pseudoaneurysm, or excision (partial or total) and graft insertion, with or without patch graft; for aneurysm, pseudoaneurysm, and associated occlusive disease, common femoral artery (profunda femoris, superficial femoral) | Repair of aneurysm or occlusive disease | Non-Endovascular |
| 35151 | 35151 Direct repair of aneurysm, pseudoaneurysm, or excision (partial or total) and graft insertion, with or without patch graft; for aneurysm, pseudoaneurysm, and associated occlusive disease, popliteal artery | Repair of aneurysm or occlusive disease | Non-Endovascular |
| 34830 | 34830 Open repair of infrarenal aortic aneurysm or dissection, plus repair of associated arterial trauma, following unsuccessful endovascular repair; tube prosthesis | Repair of aneurysm or occlusive disease | Non-Endovascular |
| 35002 | 35002 Direct repair of aneurysm, pseudoaneurysm, or excision (partial or total) and graft insertion, with or without patch graft; for ruptured aneurysm, carotid, subclavian artery, by neck incision | Repair of aneurysm or occlusive disease | Non-Endovascular |
| 35082 | 35082 Direct repair of aneurysm, pseudoaneurysm, or excision (partial or total) and graft insertion, with or without patch graft; for ruptured aneurysm, abdominal aorta | Repair of aneurysm or occlusive disease | Non-Endovascular |
| 35092 | 35092 Direct repair of aneurysm, pseudoaneurysm, or excision (partial or total) and graft insertion, with or without patch graft; for ruptured aneurysm, abdominal aorta involving visceral vessels (mesenteric, celiac, renal) | Repair of aneurysm or occlusive disease | Non-Endovascular |
| 35103 | 35103 Direct repair of aneurysm, pseudoaneurysm, or excision (partial or total) and graft insertion, with or without patch graft; for ruptured aneurysm, abdominal aorta involving iliac vessels (common, hypogastric, external) | Repair of aneurysm or occlusive disease | Non-Endovascular |
| 35142 | 35142 Direct repair of aneurysm, pseudoaneurysm, or excision (partial or total) and graft insertion, with or without patch graft; for ruptured aneurysm, common femoral artery (profunda femoris, superficial femoral) | Repair of aneurysm or occlusive disease | Non-Endovascular |
| 35045 | 35045 Direct repair of aneurysm, pseudoaneurysm, or excision (partial or total) and graft insertion, with or without patch graft; for aneurysm, pseudoaneurysm, and associated occlusive disease, radial or ulnar artery | Repair of aneurysm or occlusive disease | Non-Endovascular |
| 37205 | 37205 Transcatheter placement of an intravascular stent(s), (except coronary, carotid, and vertebral vessel), percutaneous; initial vessel | Stent placement | Endovascular |
| 37206 | 37206 Transcatheter placement of an intravascular stent(s), (except coronary, carotid, and vertebral vessel), percutaneous; each additional vessel (List separately in addition to code for primary procedure) | Stent placement | Endovascular |
| 37207 | 37207 Transcatheter placement of an intravascular stent(s), (non-coronary vessel), open; initial vessel | Stent placement | Endovascular |
| 37208 | 37208 Transcatheter placement of an intravascular stent(s), (non-coronary vessel), open; each additional vessel (List separately in addition to code for primary procedure) | Stent placement | Endovascular |
| 35321 | 35321 Thromboendarterectomy, with or without patch graft; axillary-brachial | Thromboendarterectomy | Non-Endovascular |
| 35331 | 35331 Thromboendarterectomy, with or without patch graft; abdominal aorta | Thromboendarterectomy | Non-Endovascular |
| 35341 | 35341 Thromboendarterectomy, with or without patch graft; mesenteric, celiac, or renal | Thromboendarterectomy | Non-Endovascular |
| 35351 | 35351 Thromboendarterectomy, with or without patch graft; iliac | Thromboendarterectomy | Non-Endovascular |
| 35355 | 35355 Thromboendarterectomy, with or without patch graft; iliofemoral | Thromboendarterectomy | Non-Endovascular |
| 35371 | 35371 Thromboendarterectomy, with or without patch graft; common femoral | Thromboendarterectomy | Non-Endovascular |
| 35372 | 35372 Thromboendarterectomy, with or without patch graft; deep (profunda) femoral | Thromboendarterectomy | Non-Endovascular |
| 35381 | 35381 Thromboendarterectomy, with or without patch graft; femoral and/or popliteal, and/or tibioperoneal | Thromboendarterectomy | Non-Endovascular |
| 35452 | 35452 Transluminal balloon angioplasty, open; aortic | Transluminal Balloon Angioplasty | Endovascular |
| 35454 | 35454 Transluminal balloon angioplasty, open; iliac | Transluminal Balloon Angioplasty | Endovascular |
| 35456 | 35456 Transluminal balloon angioplasty, open; femoral-popliteal | Transluminal Balloon Angioplasty | Endovascular |
| 35458 | 35458 Transluminal balloon angioplasty, open; brachiocephalic trunk or branches, each vessel | Transluminal Balloon Angioplasty | Endovascular |
| 35459 | 35459 Transluminal balloon angioplasty, open; tibioperoneal trunk and branches | Transluminal Balloon Angioplasty | Endovascular |
| 35470 | 35470 Transluminal balloon angioplasty, percutaneous; tibioperoneal trunk or branches, each vessel | Transluminal Balloon Angioplasty | Endovascular |
| 35472 | 35472 Transluminal balloon angioplasty, percutaneous; aortic | Transluminal Balloon Angioplasty | Endovascular |
| 35473 | 35473 Transluminal balloon angioplasty, percutaneous; iliac | Transluminal Balloon Angioplasty | Endovascular |
| 35474 | 35474 Transluminal balloon angioplasty, percutaneous; femoral-popliteal | Transluminal Balloon Angioplasty | Endovascular |
| 35475 | 35475 Transluminal balloon angioplasty, percutaneous; brachiocephalic trunk or branches, each | Transluminal Balloon Angioplasty | Endovascular |
| 35476 | 35476 Transluminal balloon angioplasty, percutaneous; venous | Transluminal Balloon Angioplasty | Non-Endovascular |
| 35493 | 35493 Transluminal peripheral atherectomy, percutaneous; femoral-popliteal | Transluminal Peripheral Atherectomy | Endovascular |
| 37607 | 37607 Ligation or banding of angioaccess arteriovenous fistula | Vessel ligation | Non-Endovascular |
| 37609 | 37609 Ligation or biopsy, temporal artery | Vessel ligation | Non-Endovascular |
| 37617 | 37617 Ligation, major artery (eg, post-traumatic, rupture); abdomen | Vessel ligation | Non-Endovascular |
| 37618 | 37618 Ligation, major artery (eg, post-traumatic, rupture); extremity | Vessel ligation | Non-Endovascular |
