# Supplementary material for: Nutritional status and out-of-hospital mortality in vascular surgery patients
Source: PLoS One. 2022 Jul 21;17(7):e0270396. doi: 10.1371/journal.pone.0270396 (PMC9302752; doi:10.1371/journal.pone.0270396)
Supplement: S1 Table — (DOCX) [file pone.0270396.s003.docx]

**Supplemental Table 1: Characteristics of Vascular Surgery Procedure Code Categories stratified by 90-Day Post Discharge Mortality^a^ in the Analytic Cohort (n=4432)**

| **Procedure Code Category** | Alive^a^ | Expired | Total | P-value |
| --- | --- | --- | --- | --- |
| N | **4,271** | **161** | **4,432** |  |
| Arteriovenous Fistula-No.(%) | **31 (1)** | **2 (1)** | **33 (1)** | **0.45** |
| Decompression-No.(%) | **181 (4)** | **3 (2)** | **184 (4)** | **0.14** |
| Thromboendarterectomy-No.(%) | **119 (3)** | **7 (4)** | **126 (3)** | **0.24** |
| Arterial Bypass-No.(%) | **453 (11)** | **15 (9)** | **468 (11)** | **0.60** |
| Embolectomy Or Thrombectomy-No.(%) | **131 (3)** | **10 (6)** | **141 (3)** | **0.026** |
| Graft Excision-No.(%) | **20 (0)** | **3 (2)** | **23 (1)** | **0.016** |
| Major Amputation-No.(%) | **327 (8)** | **23 (14)** | **350 (8)** | **0.02** |
| Minor Amputation-No.(%) | **217 (5)** | **10 (6)** | **227 (5)** | **0.52** |
| Blood Vessel Repair-No.(%) | **930 (22)** | **14 (9)** | **944 (21)** | **<0.001** |
| Stent Placement-No(%) | **650 (15)** | **21 (13)** | **671 (15)** | **0.45** |
| Transluminal Balloon Angioplasty-No.(%) | **578 (14)** | **27 (17)** | **605 (14)** | **0.24** |
| Vessel Ligation-No.(%) | **74 (2)** | **3 (2)** | **77 (2)** | **0.90** |
| Artery Exposure/Exploration-No.(%) | **394 (9)** | **17 (11)** | **411 (9)** | **0.57** |
| Transluminal Peripheral Atherectomy-No.(%) | **23 (1)** | **0 (0)** | **23 (1)** | **0.35** |
| Repair of Aneurysm-No.(%) | **445 (10)** | **15 (9)** | **460 (10)** | **0.65** |
| Endovascular-No.(%) | **2013 (47)** | **85 (53)** | **2098 (47)** | **0.16** |

Note: a. Expired within 90-days following hospital discharge**.** No.(%) refers to the number of procedures performed (proportion of procedures per patient in each group). The percentages do not add up to 100 as each patient may have more than one CPT coded procedure.
