# Supplementary material for: Nutritional status and out-of-hospital mortality in vascular surgery patients
Source: PLoS One. 2022 Jul 21;17(7):e0270396. doi: 10.1371/journal.pone.0270396 (PMC9302752; doi:10.1371/journal.pone.0270396)
Supplement: S2 Table — (DOCX) [file pone.0270396.s004.docx]

| **Procedure Code Category** | **Nutrition Status** | | | |
| --- | --- | --- | --- | --- |
|  | **No malnutrition** | **At risk for malnutrition** | **Non-specific malnutrition** | **Specific protein-calorie malnutrition** |
| N | **3,819** | **215** | **351** | **47** |
| Arteriovenous Fistula-No.(%) | **25 (1)** | **3 (1)** | **3 (1)** | **2 (4)** |
| Decompression-No.(%) | **143 (4)** | **13 (6)** | **25 (7)** | **3 (6)** |
| Thromboendarterectomy-No.(%) | **111 (3)** | **3 (1)** | **11 (3)** | **1 (2)** |
| Arterial Bypass-No.(%) | **418 (11)** | **16 (7)** | **29 (8)** | **5 (11)** |
| Embolectomy Or Thrombectomy-No.(%) | **119 (3)** | **9 (4)** | **12 (3)** | **1 (2)** |
| Graft Excision-No.(%) | **17 (0)** | **1 (0)** | **5 (1)** | **0 (0)** |
| Major Amputation-No.(%) | **305 (8)** | **13 (6)** | **26 (7)** | **6 (13)** |
| Minor Amputation-No.(%) | **206 (5)** | **9 (4)** | **10 (3)** | **2 (4)** |
| Blood Vessel Repair-No.(%) | **862 (23)** | **31 (14)** | **50 (14)** | **1 (2)** |
| Stent Placement-No(%) | **647 (17)** | **9 (4)** | **11 (3)** | **4 (9)** |
| Transluminal Balloon Angioplasty-No.(%) | **577 (15)** | **13 (6)** | **11 (3)** | **4 (9)** |
| Vessel Ligation-No.(%) | **61 (2)** | **4 (2)** | **10 (3)** | **2 (4)** |
| Artery Exposure/Exploration-No.(%) | **334 (9)** | **26 (12)** | **47 (13)** | **4 (9)** |
| Transluminal Peripheral Atherectomy-No.(%) | **23 (1)** | **0 (0)** | **0 (0)** | **0 (0)** |
| Repair of Aneurysm-No.(%) | **416 (11)** | **7 (3)** | **36 (10)** | **1 (2)** |
| Endovascular-No.(%) | **1806 (47)** | **115 (53)** | **157 (45)** | **20 (43)** |

**Supplemental Table 2. Characteristics of** **Vascular Surgery Procedure Code Categories stratified by Nutritional Status in the Analytic Cohort (n=4432)**

Note: No.(%) refers to the number of procedures performed (proportion of procedures per patient in each Nutrition Status group). The percentages do not add up to 100 as each patient may have more than one procedure in a procedure group.
